# Sensory, Microbiological and Physicochemical Characterisation of Functional Manuka Honey Yogurts Containing Probiotic *Lactobacillus reuteri* DPC16

**DOI:** 10.3390/foods9010106

**Published:** 2020-01-19

**Authors:** Anand Mohan, Joshua Hadi, Noemi Gutierrez-Maddox, Yu Li, Ivanhoe K. H. Leung, Yihuai Gao, Quan Shu, Siew-Young Quek

**Affiliations:** 1 School of Chemical Sciences, University of Auckland, Private Bag 92019, Victoria Street West, Auckland 1142, New Zealand; amoh440@aucklanduni.ac.nz (A.M.); jhad391@aucklanduni.ac.nz (J.H.); yli648@aucklanduni.ac.nz (Y.L.); i.leung@auckland.ac.nz (I.K.H.L.); 2 School of Applied Sciences, Auckland University of Technology, Private Bag 92006, Auckland 1142, New Zealand; noemi.gutierrezmaddox@aut.ac.nz; 3 Bioactives Research New Zealand Limited, 465-467 Khyber Pass Road, Newmarket, Auckland 1023, New Zealand; yhgao@ganopoly.com (Y.G.); marsh.shu@gmail.com (Q.S.)

**Keywords:** consumer acceptance, probiotic, prebiotic, manuka honey, yogurt, physico-chemical, ^1^H-^13^C HSQC NMR, fermentation metabolites

## Abstract

Consumer acceptance of synbiotics, which are synergistic combinations of probiotics and their prebiotic substrates, continues to expand in the functional food category. This research aimed at evaluating the effect of antibacterial manuka honey on the probiotic growth and sensory characteristics of potentially synbiotic yogurts manufactured with *Lactobacillus reuteri* DPC16. Probiotic viable count in yogurts with 5% *w*/*v* Manuka honey (Blend, UMF^TM^ 18^+^, AMF^TM^ 15^+^ and AMF^TM^ 20^+^) was evaluated by the spread plate method over the refrigerated storage period of three weeks. A panel of 102 consumers preferred the yogurt made with invert syrup over the manuka honey variants, and the unsweetened control was least liked overall. Invert syrup yogurt was also the most effective in promoting the growth of the probiotic lactobacilli. However, the honey-sweetened yogurts had a more favourable fermentation metabolite profile, especially the lactic and propionic acids, as estimated by nuclear magnetic resonance (NMR) analyses. The probiotic counts in AMF^TM^ 15^+^ manuka honey yogurt (7 log cfu/mL) were significantly higher than the other honey yogurt types (Manuka Blend and UMF^TM^ 18^+^) and above the recommended threshold levels. The combination thus can be developed as a synbiotic functional food by further improving the sensory and physicochemical properties such as texture, apparent viscosity and water holding capacity.

## 1. Introduction

Functional foods with probiotics are often supplemented with co-cultures, such as yogurt starters, to manufacture products with acceptable sensory characteristics and consumer acceptance. Lactic acid bacteria (LAB) includes the yogurt starter cultures (*Lactobacillus delbrueckii* ssp. *bulgaricus* and *Streptococcus thermophilus*), and probiotic species mostly belonging to the *Lactobacillus* and *Bifidobacterium* genera. It is important to note that the probiotic bacterial species are different from the starter cultures commonly used for fermenting milk into yogurts. The probiotics have superior survivability in low pH in the stomach and high bile salt concentrations in the small intestine and are thus able to reach the large intestine to benefit the health of the host in numerous ways [1,2]. For a beneficial health effect on the host, the recommended minimum dosages of probiotics reported in the literature range from 6 to 7 log colony forming units (cfu) per millilitre (mL) of the sample under consideration [3,4]. Furthermore, maintaining the viable counts at the efficacious levels during the storage period is one of the major technological challenges for probiotics incorporation in functional foods [2,3].

Probiotics are known to improve the host digestive and immune systems as their ‘core benefits’ but the distinctive health benefits, such as the production of bioactives, are strain-specific [1]. The probiotic *Lactobacillus reuteri* demonstrated excellent functional health effects in earlier studies [5,6]. The probiotic strain utilised in our study, *Lactobacillus reuteri* DPC16, has proven antimicrobial activity against certain pathogens by the mechanisms of producing reuterin and organic acids [7,8]. Previous studies, using immobilised cells, have reported low death rate on passage through the simulated gastrointestinal tract [9,10] and the inhibition of pathogen adhesion to epithelial cells [7,11].

The selection of the correct probiotic strains, along with the consumption of prebiotics, ensures a subsequent favourable microbial balance in the gut [2,3]. Prebiotics were initially defined as non-digestible and fermentable carbohydrates, which allow the lactobacilli and bifidobacteria in the gut microflora to be nourished [12]. This not only increases the probiotic biomass in the large intestine but also produces beneficial fermentation metabolites, such as short-chain fatty acids (SCFA), which are known to contribute positively to the holistic physiological benefits attributable to the gut microbiome. The definition of prebiotics was recently updated as substrates, including non-carbohydrate based, which are selectively consumed by the inhabiting microorganisms and thus conferring health benefits to the host [13]. Moreover, the efficacy of probiotics against the pathogenic bacteria is ascribed to various factors, including the production of the organic acids and certain antibacterial substances, for example, reuterin by *Lactobacillus reuteri* [6].

Oligosaccharides in honey (approximately 5–10%) are mostly non-digestible and are thus also a preferred substrate for intestinal probiotic bacteria [14,15], which in addition to its inherent antimicrobial activity makes honey a potential prebiotic candidate [2]. Furthermore, the distinctive non-peroxide antibacterial effect of manuka honey has been attributed to high levels of methylglyoxal [16], among other factors, and is represented by Unique Manuka Factor (UMF^TM^), or Active Manuka Factor (AMF^TM^), or Methylglyoxal (MGO^TM^), on the product label. The research on manuka honey has primarily focused on areas such as the antibacterial action of the components, the well-established wound-healing effect, and the possible mitigation of antibiotic resistance, but not so much on its efficacy and health impacts in typical food systems [2].

The organic acid characterisation in fermented foods such as yogurts is crucial for determining its sensory properties, health effects, and as an indicator of the bacterial activity of the cultures and probiotics [17]. Identification and quantification of volatile organic acids from food and biological materials are generally accomplished using gas chromatography (GC-FID/GC-MS), while liquid chromatography (HPLC) is a suitable method for non-volatile SCFAs [18]. Moreover, nuclear magnetic resonance (NMR) investigation is non-invasive, entailing minimal sample preparation, and thus has high repeatability in results for the complex sample matrices [19]. ^1^H-^13^C heteronuclear single quantum correlation (HSQC) NMR quantification of the whole milk constituents was first reported by Hu et al. [20], and the present study has further optimised the method to estimate the organic acid metabolites produced in yogurt.

When carefully selected, a prebiotic reinforces the probiotic viability in the gut, and this combination is termed as synbiotic [21]. Prebiotics incorporated along with probiotics, although a healthier option for consumers, may affect the acceptability of the product. Thus, the aim of the study was to evaluate a potentially synbiotic functional food with the probiotic lactobacilli strain, by evaluating the microbiological, physicochemical, textural, and sensory properties of yogurts with three different varieties of Manuka honey (UMF^TM^, AMF^TM^ and Manuka Blend). It was hypothesised that a probiotic growth-promoting effect from predominantly antibacterial manuka honey would result in a valuable functional food. To the best of the authors’ knowledge, this is the first study that has utilised manuka honey as yogurt sweetener for studying its potentially prebiotic effect that included the release of the beneficial SCFA metabolites.

## 2. Materials and Methods

### 2.1. Microbial Strains, Media, Ingredients and Chemicals

Probiotic *Lactobacillus reuteri* DPC16 culture was kindly provided by Bioactives Research New Zealand. Yogurt starter culture Lyofast Y 450B (*Lactobacillus delbrueckii* ssp. *bulgaricus* and *Streptococcus thermophilus*) was obtained from SACCO System, Italy. Local purchases were made for invert syrup (IS) and manuka honey with different antibacterial gradings: Drapac Manuka Honey Blend (MB), Comvita Manuka Honey UMF^TM^ 18^+^ (UMF18), and Drapac DrKiwi AMF^TM^ 15^+^ (AMF15) and AMF 20^+^ (AMF20). Yogurts for the sensory and physicochemical analysis were prepared with fresh full-cream Anchor Blue milk bought from a supermarket. Microbiological enumeration and NMR analyses were conducted with yogurts made from nil-fat UHT milk. Difco^TM^ MRS (de Mann, Rogosa and Sharpe) broth and agar were utilised as media for bacterial growth and plate counts respectively. Analytical standards for lactic acid, acetic acid, butyric acid, propionic acid, isobutyric acid and succinic acid were obtained from Sigma Aldrich (Christchurch, New Zealand).

### 2.2. Probiotic Pellet Preparation

The method was adapted from Zhao et al. [11] with modifications. Briefly, 2 mL of *L. reuteri* DPC16 strain was subcultured and inoculated into 100 mL of MRS broth and incubated at 35 °C for 48 h. After the incubation period, 40 mL of the MRS broth containing probiotic culture was poured into a centrifuge tube and centrifuged at 7000× *g* for 10 min at 4 °C. The supernatant was carefully removed, and the probiotic pellets at the bottom of the tubes were washed with saline water (0.5% *w*/*v*) by shaking thoroughly. The washing procedure was repeated twice along with centrifugation under the same conditions as mentioned above. The probiotic pellets thus obtained were suspended in milk and refrigerated at 4 °C until yogurt preparation on the day.

### 2.3. Probiotic Yogurt Preparation

Food grade yogurts for sensory evaluation and physicochemical analyses were prepared in the Food Science Laboratory at the University of Auckland. Milk (6 L) was heated to 95 °C for 5 min and cooled to 40 °C, at which point the commercial freeze-dried starter culture (10 mg/L) was added and thoroughly mixed. This was followed by the addition of probiotic pellets prepared as explained above to achieve a minimum viable count of 7 log cfu/mL in all the samples. Initial incubation was done at 35 °C for 1 h to ensure uniform distribution of the microbes. Honey/Invert syrup (5% *w*/*v*) was then added in the five equally divided batches respectively. Final incubation was done in the respective containers until a pH of 4.5–4.6 was achieved. Samples were cooled immediately after fermentation and refrigerated overnight for analysis on days 1, 7, 14 and 21, respectively. A similar procedure, except the heating step, was followed for the yogurts prepared with UHT milk for the microbiological and NMR analyses.

### 2.4. Bacteria Enumeration

Total viable counts of *Lactobacillus reuteri* DPC16 were enumerated by the spread plate method on MRS agar. Yogurt samples (10 mL) were transferred into 90 mL sterile peptone water to obtain 1:10 dilution. Subsequently, 1 mL of this solution was transferred to 9 mL peptone water and serially diluted seven times. Each dilution was plated on MRS agar and incubated at 37 °C for 48 h. Incubations were done in a 5% CO_2_ atmosphere to stimulate the probiotic lactobacilli growth. In the present study, it was ensured that the enumeration conditions were not favourable for the growth of yogurt starter culture bacteria: *Lactobacillus delbrueckii* ssp. *bulgaricus* requires a pH 5.2 in MRS agar (45 ℃ for more than 72 h) and *Streptococcus thermophilus* grows aerobically in *S. thermophilus* agar (37 ℃ for 24 h) [22]. Furthermore, in our study, the probiotic *Lactobacillus reuteri* DPC16 formed large (2 mm), milky-white smooth colonies that would be easily distinguishable from the smaller (1 mm), white, cottony-rough irregular colonies of *Lactobacillus delbrueckii* ssp. *bulgaricus* [23]. Total viable counts were recorded on plates that contained colonies ranging from 30 to 300 and were expressed as the log cfu/mL of yogurt.

### 2.5. Organic Acids Quantification

Quantification of the organic acid metabolites was conducted by ^1^H-^13^C HSQC NMR. Yogurt samples were first diluted with Type I water and D_2_O (2.25:2.25:0.5—Yogurt:Type I water:D_2_O ratio) and introduced in 5 mm NMR tubes (500 μL total volume). Analytical standards of pure organic compounds used for the quantification were also diluted in D_2_O in the same ratio (9:1). The experiments were performed on a Bruker Avance III 400 MHz spectrometer at a temperature of 300K (27 ℃). Water suppression was done using the excitation sculpting method [24]. Pulse calibration was performed using the Bruker ‘pulsecal’ technique [25].

### 2.6. Sensory Evaluation

All yogurt samples, after refrigerated storage overnight, were subjected to hedonic sensory evaluation (consumer acceptance test) by a total of 102 participants. The untrained panellists consisted mostly of university students and a few staff members, who were consumers of yogurts with different frequencies. Prior authorisation from the University of Auckland Human Participants Ethics Committee (UAHPEC) was granted on 30 June 2017 for three years (reference number 01922). The researchers obtained informed consent from each of the participants immediately before the trials. A 7-point hedonic scale with ratings from “extreme dislike” on the left (a score of 1), “neither like nor dislike” in the middle (a score of 4) and “extreme like” on the right (a score of 7) was employed. The sensory attributes evaluated by the consumer panellists were colour, appearance, mouthfeel (thickness), smoothness, sweetness, sourness and overall acceptability. Randomisation was performed by applying the Latin-squares method to minimise any left-over effects from the preceding sample, such as aftertaste, flavour or similar psychological influences. This technique was adapted from Wakeling and MacFie [26], which entails ensuring that every yogurt sample was equally likely to be presented before every other. No information about the sample types was provided to the panellists to prevent any biases.

### 2.7. Physicochemical Analysis

The acidity of each yogurt sample was measured with a Sartorius PB-10 pH meter. The colour on the surface of the yogurt samples, as quantified using a Minolta Chroma Meter CR-300 (Minolta Co. Ltd., Japan), was reported in three distinct coordinates of L* (lightness; white: 100, black: 0), a* (red: +, green: −) and b* (yellow: +, blue: −).

Viscosity analysis was conducted at a temperature of 14.8 ± 1 °C using a Brookfield digital rotational viscometer (model DV-III+, Brookfield Engineering Laboratories, Middleboro, MA, USA), as per the method adapted from Isleten and Karagul-Yuceer [27]. Viscosity readings for 50 mL samples were taken at 60 and 70 s, and the mean values were calculated for the triplicates accordingly. Spindle no. 6 of the viscometer was run at 100 revolutions per minute (rpm) to achieve the torque levels recommended by the manufacturer (10–100%).

Texture analysis was performed on TA.XT.Plus (Stable Micro Systems Ltd., Surrey, UK) equipped with a 50-kg load cell. The method was adapted from Costa et al. [28], with some modifications. Briefly, 50 mL samples were subjected to compression using a rear extrusion disc (diameter 12 mm; distance 30 mm; speed 1 mm/s) placed centrally over the sample beaker to record the values of firmness (force applied for disrupting the yogurt structure formed during cold storage), consistency (the extent to which the structure of yogurt deforms before it ruptures), and cohesiveness (the property by which the structure withstands a change in its shape) [28,29].

Water holding capacity (WHC) was calculated applying the method adapted from Sert et al. [30], with certain modifications. Briefly, the samples were sheared at 1250× *g* for 70 s to disrupt the gel network and subsequently restructured for 24 h at 4 °C. After the holding period, 20 mL samples were put on a Büchner funnel lined with a strainer cloth and drained out for 30 min at ambient temperature. The initial weight of the yogurt samples (I) and the whey discarded (W) were recorded. The WHC was expressed as per Equation (1).

(1) $$\mathrm{WHC}=\frac{I-W}{I}\times 100\%$$

### 2.8. Statistical Analysis

The results for probiotic enumeration, sensory and physicochemical evaluation, and organic acids quantification were subjected to one-way ANOVA, with statistical significance (*p* < 0.05) determined by “Tukey’s b” multiple comparison test. For inferring the statistical significance, three independent replicates were prepared for bacterial counts and organic acid metabolites analyses. Each of the yogurt sample types evaluated by the consumer panel was subjected to physicochemical analysis in triplicate, the values for which are reported as mean ± standard deviation. The sensory attributes were also depicted by error bars that capture the population mean ratings in 95% confidence intervals. This was achieved for the consumer participants sample size (*n* = 102) by adjusting the standard error (SE) bars with a factor of ‘*t*’ [31].

## 3. Results and Discussion

### 3.1. Total Viable Counts

**Figure 1:**
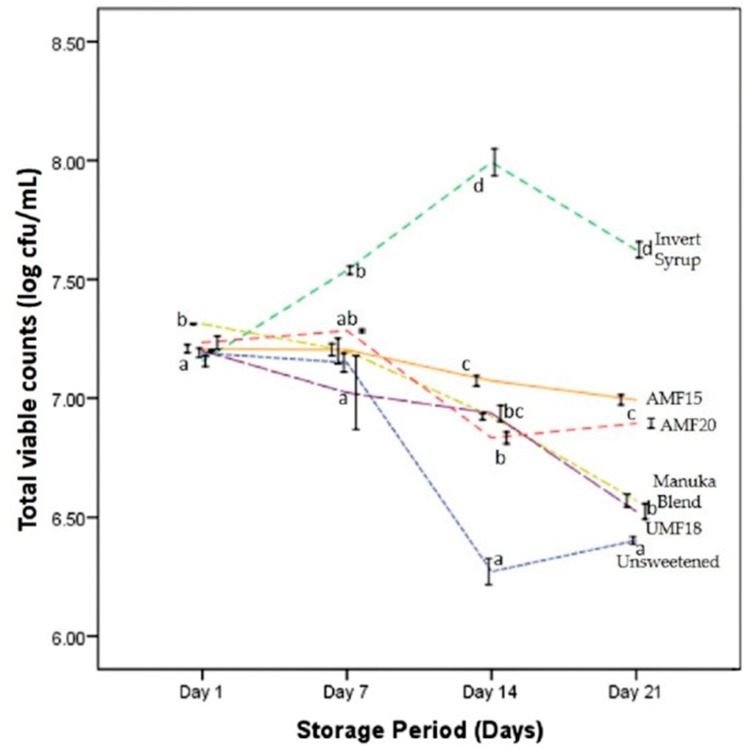
Lactic acid bacteria (LAB) counts (log colony forming units / mL) in yogurts with Manuka honey (Blend, UMF^TM^ 18^+^ and AMF^TM^ 15^+^), invert syrup, and unsweetened control, during the refrigerated storage period (21 days). Different lowercase letters indicate significant differences (*p* < 0.05) among the yogurt samples (on that particular day). Error bars represent standard error of means (±1 SE).

LAB counts at day 1 of cold storage were above 7 log cfu/mL in all the yogurts samples, with the highest being in MB (7.31 log cfu/mL). As can be seen in Figure 1, the total counts of viable probiotics decreased during the refrigerated storage period of three weeks, except in the yogurt containing invert syrup (IS). This decrease was, however, not statistically significant up to day 7 for all the honey-sweetened yogurts. In all the instances in Figure 1, nevertheless, the viable counts of the probiotics were maintained above the recommended minimum dosage of 6 log cfu/mL [3] throughout the storage period.

The addition of AMF15 manuka honey to the yogurt maintained a high probiotic survival (7.0 log cfu/mL) after the three-week refrigerated storage (4 °C). The counts were significantly (*p* < 0.01) higher than those for the MB (6.57 log cfu/mL), UMF18 (6.52 log cfu/mL) and the control sample without any added sweetener (6.4 log cfu/mL) but lower than the 7.62 log cfu/mL recorded in IS yogurts (Figure 1). All sweetened samples had higher LAB counts than the unsweetened control, but only the honey-sweetened yogurts can be considered as potential prebiotics because of the non-digestible oligosaccharide components.

The total counts for AMF20 (6.89 log cfu/mL) were not significantly different (*p* > 0.05) from AMF15. Thus, the more expensive grade of manuka honey (AMF20) was not utilised for sensory and other trials. It is pertinent to note that the survivability of the LAB in yogurts with manuka honey, which has proven antibacterial activity, was superior to the unsweetened control. As per the product label, DrKiwi AMF^TM^ Manuka honey contains the fermentation metabolites of the *Lactobacillus reuteri* DPC16, which contributes to the Active Manuka Factor (AMF^TM^) rating of the honey, and this could be one of the reasons for the honey not being inhibitory to this probiotic strain. It is, therefore, necessary to further characterise the different components of the manuka honey and evaluate the physiological effects in in vivo studies. This will then, according to the recently broadened definition [13], fully establish a prebiotic effect. There is no prior study that had utilised this particular strain with manuka honey, and thus, this potential synbiotic combination is unexplored so far.

Previous research has demonstrated that adding honey at a concentration of 5% *w*/*v* in yogurts or fermented milk to be optimal for the growth of probiotics and starter culture bacteria and for their survival during refrigerated storage period [15,32]. Earlier, Curda and Plocková [33] found that honey above a concentration of 5% (and up to 10% *w*/*v* that was studied) had an inhibitory effect on *Lactobacillus acidophilus* A92 and a starter culture consisting of *Lactococus lactis* ssp. *lactis*. Our preliminary trials concluded that 5% (and not 7.5%) was the optimum level of honey to obtain the desirable sensory and physiochemical attributes, such as pH and visual syneresis. A study by Sert et al. [30] had previously concluded that the optimal sweetness in sunflower honey yogurts without probiotics was at 4% (compared with 2% or 6% *w*/*v*). Riazi and Ziar [34] noted that the growth of a probiotic *Bifidobacterium* strain was enhanced in a similar associated culture system with the yogurt starters (as in the present study). The researchers concluded that the combination with 5% (*w*/*v*) honey concentration was ideal for commercial yogurt production. However, their study compared the growth of probiotic bifidobacteria strains (not lactobacilli) with various uni- and poly-floral honey varieties.

A very similar survival-death curve to Figure 1 was recently published by Machado et al. [4] for probiotic *Lactobacillus acidophilus*. The study reported no statistically significant difference in the viable counts of the probiotic in goat milk yogurt with different stingless bee honey concentrations (5%, 10% and 15% *w*/*v*) throughout the four-week storage period. The counts were, nonetheless, significantly higher than the unsweetened controls from the second week onwards. No mono- or di- saccharide (sucrose or invert syrup) was employed as a control in the two studies mentioned above. Honey can be considered as essentially an invert sugar solution based on its sugar composition [30], containing relatively minor amounts of additional sucrose and oligosaccharides. However, to the best of the authors’ knowledge, there is no similar study in the literature to date that has utilised invert syrup as a control for honey-sweetened fermented milk or yogurts.

Popa and Ustunol [17] reported a significantly higher growth for *Lactobacillus acidophilus* milk in sucrose (9.16 log cfu/mL) than inulin (8.56 log cfu/mL), but not with the different kinds of honey (8.91–9.06 log cfu/mL). In a previous study from the same research group, Chick et al. [32] found no significant difference in the growth-promoting effect of sucrose, fructose and honey (5% *w*/*v* in reconstituted milk) with the unsweetened control. The yogurt starter culture in the study was co-cultured with the probiotic *Lactobacillus acidophilus* or *Bifidobacterium bifidum*. Several studies have published a superior probiotic growth for bifidobacteria compared to lactobacilli [32,35,36]. This has been attributed to the fact that low degree of polymerisation (low-DP) oligosaccharides, such as those found in honey or inulin, are a preferred substrate for bifidobacteria [14,37].

### 3.2. Organic Acid Production

**Figure 2:**
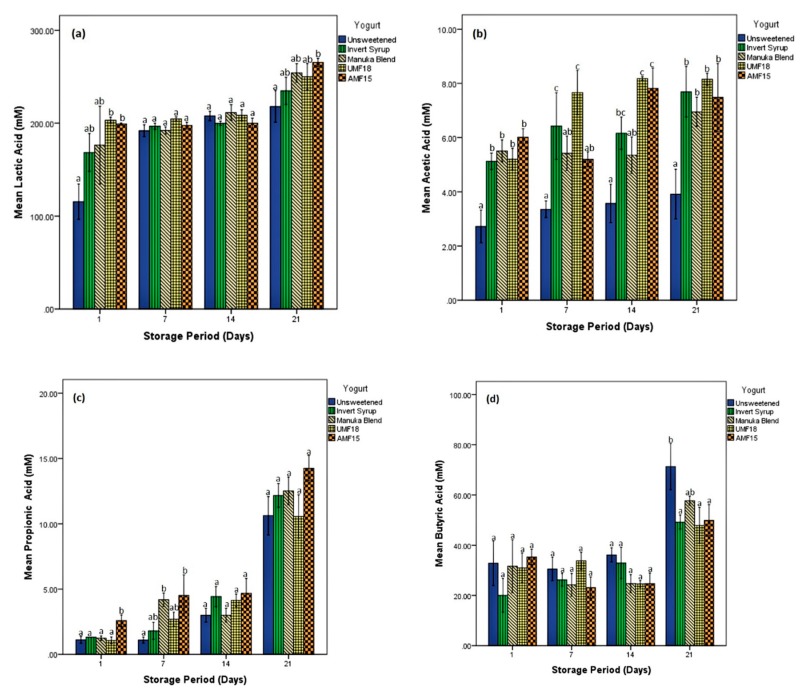
Concentration (mM) of (**a**) lactic acid (**b**) acetic acid (**c**) propionic acid and (**d**) butyric acid produced in yogurts with three different types of Manuka honey (Blend, UMF^TM^ 18^+^ and AMF^TM^ 15^+^) invert syrup, and unsweetened control, over the refrigerated storage period (21 days). Different lowercase letters indicate significant differences (*p* < 0.05) among the yogurt samples (on that particular day). Error bars represent standard error of means (±1 SE).

The results obtained using NMR quantification are shown in Figure 2. Lactic acid (Figure 2a) was the major organic acid fermentation metabolite, followed by the shorter chain fatty acids: acetic (Figure 2b), propionic (Figure 2c) and butyric (Figure 2d). The initial levels of these organic acids in milk and some of the other metabolites produced in the yogurts, such as isobutyric and succinic acids, were below the detection threshold of ^1^H-^13^C NMR HSQC. It is interesting to note that the concentrations of the organic acids increased noticeably on day 21, which had also indicated an onset of visual spoilage on inspection. The increase was observed for lactic, butyric and propionic but not acetic acid.

Yogurt starter cultures and probiotics produce lactate by fermenting the lactose in milk. Compared with the other SCFAs, lactic acid (LA) imparts the desirable sensory attributes in fermented dairy foods such as yogurt [3]. LA generated by honey-sweetened yogurts was prominently more than the unsweetened sample, especially on days 1 and 21 (Figure 2a). Furthermore, LA from yogurt containing AMF15 (199.1 mM) was significantly (*p* < 0.05) more than that of the unsweetened control (115.5 mM) on day 1, and also on day 21 (265.4 mM and 217.9 mM, respectively). LA production in the IS yogurt was only marginally (*p* > 0.05) higher than the unsweetened control yogurt. Chick et al. [32] reported a significant difference in lactic acid generated by *Bifidobacterium bifidum* with clover honey (26 mM) compared with sucrose (10.4 mM), fructose (7.2 mM) and the unsweetened control (4.6 mM). However, in the same study, no significant difference was found in lactic acid production when the probiotic was lactobacilli (*Lactobacillus acidophilus*). A more recent publication by the research group [17], reported that lactic acid yielded with sourwood honey (355.2 mM) and *Lactobacillus acidophilus* was significantly higher than that with high-fructose corn syrup (HFCS) (331.4 mM), sucrose (337.6 mM) and unsweetened control (313.9 mM).

In the study by Popa and Ustunol [17], a significantly high concentration of acetic acid (59.78 mM) was reported with a potentially synbiotic combination of sourwood honey and *Bifidobacterium bifidum* Bf-1. The over-production of acetic acid (AA) yields an inappropriate vinegary flavour. Thus, the outcome in the present study is desirable from a consumer sensory perspective since not more than 8.2 mM AA (UMF18) was detected in any of the samples, even after 21 days (Figure 2b). The AA production for all the sweetened yogurts, including the IS, was more than the control without any sweetener. The difference in AA produced was statistically significant (*p* < 0.05) on days 1 and 21. Haddadin et al. [38] reported a significant increase in acetic, propionic and butyric acid generated with three Jordan honey (7.5% *w*/*v* in reconstituted milk) substrates compared to the unsweetened control after 16 h of fermentation. However, the probiotic strains utilised were *Bifidobacterium infantis* and *Lactobacillus acidophilus*, and the LA content was not directly measured in that study.

The production of propionic acid (PA) steadily increased with the storage days (Figure 2c). AMF15 yogurt produced higher PA than all other yogurts, with the difference being significantly (*p* < 0.05) higher than the unsweetened sample on day 1 (2.6 mM vs. 1.1 mM) and day 7 (4.51 mM vs. 1.08 mM). A highly cited paper by Donkor et al. [3] reported that the production of PA was enhanced by lower concentrations (0.5–1.0%) of resistant starch (amylose maize) but not inulin. The study was conducted in set-type yogurts with either *Lactobacillus acidophilus* or *Lactobacillus casei* as the probiotic strain. In concurrence with our results, the researchers also reported that production of both LA and AA significantly increased during the storage period for both their prebiotics. Furthermore, the LA concentration in their study was influenced by the prebiotic type, with inulin yielding more of the metabolite than amylose maize starch. The AA concentration in their study, however, was not affected by either the nature of the prebiotic or its level.

As can be seen in Figure 2d, the average butyric acid (BA) production (20 to 71.3 mM) was lower only than LA as quantified in Figure 2a (115.5 to 265.4 mM), for all samples during the storage weeks. Moreover, in contrast to the trend observed for the three organic acids mentioned earlier, the unsweetened yogurt yielded higher amounts of BA than the sweetened yogurts. The produced BA in the unsweetened control (71.3 mM) was significantly (*p* < 0.05) higher than that in the sweetened yogurts (IS, UMF18 and AMF15) on day 21. Vaseji et al. [39] reported significantly higher concentrations of BA in probiotic yogurts (without added honey or prebiotics) than the regular yogurts that were cultured using only *Lactobacillus delbrueckii* ssp. *bulgaricus* and *Streptococcus thermophilus*. Interestingly, the probiotic yogurt that had both the *Bifidobacterium* and *Lactobacillus acidophilus* strains produced even more BA than the one containing only *Lactobacillus acidophilus* as the additional probiotic. In the present study, therefore, honey addition (notably the AMF15) to yogurts enhanced the organic acid metabolite profile except for BA production, which is thus more likely to be a factor of the type of probiotic strain (bifidobacteria or lactobacilli) rather than the prebiotic substrate.

### 3.3. Consumer Acceptance Ratings

The overall acceptance ratings of samples supplemented with sweeteners were significantly (*p* < 0.05) greater than those of the unsweetened control sample, with IS and MB having a high overall acceptability score above 5.0 on the 7-point scales (Table 1). The sensory rating data are also reproduced in Appendix A, with error bars capturing the population mean ratings in 95% confidence intervals. No statistically significant differences in the consumer ratings among sweetened yogurts were observed for the attributes of colour, appearance, mouthfeel and smoothness. Overall acceptance was, thus, likely dependent on the consumer preference of the sweetness and sourness levels in the yogurts. This concurs with earlier research on predicting consumer acceptability of yogurt, which concluded that an optimally high sweetness (as evaluated by a trained descriptive panel and, interestingly, not analytical instruments) in flavoured yogurt yields higher consumer acceptability [40].

As is evident in Table 1, the unsweetened yogurt was least liked for the sweetness attribute (3.51 ± 1.46), and its acceptance rating was significantly (*p* < 0.01) lower than all the samples. This could be attributed to the powerful hedonic appeal of sweetness amongst the consumers, which was corroborated in previous studies that reported an enhanced preference for sweetened yogurts [40,41,42]. The sweeteners also improve the mouthfeel, texture, viscosity and water-holding capacity of yogurts [43].

In a recent report by Machado et al. [4], probiotic (*Lactobacillus acidophilus*) goat milk yogurts with stingless bee honey had significantly higher overall acceptability than the unsweetened controls, with the most preferred formulations being at the high honey concentrations (10 and 15 % *w*/*v*). This aspect was in contrast to our preliminary trials in which the addition of honey at a higher level of 7.5% *w*/*v* was detrimental to the visual sensory attributes since the pH did not reach the required levels (4.5–4.6) during the incubation period. This was possibly due to the pronounced antibacterial effects of manuka honey at higher dosages. Furthermore, because of the considerably high cost of manuka honey, it was imperative to optimise the honey percentage that had sensorial acceptance and facilitated probiotic growth. The high sensory preference for yogurts with added honey was attributed by Machado et al. [4] to the positive effect on certain physicochemical properties, such as increasing the viscosity, water holding capacity and the total solid content and enhancing the colour and consistency of the yogurt sample. Furthermore, the sweetness and additional flavouring components of the stingless bee honey had effectively masked the high acidity developed during fermentation.

The higher overall acceptance ratings of the yogurts sweetened with IS (5.32 ± 1.06) and MB (5.05 ± 1.24) can be attributed to the higher consumer acceptance of their sweetness and sourness perception. This was further corroborated by several additional comments by the consumer panel suggesting that the unsweetened samples needed to be sweeter or less sour. As per the subjective observations of the participants, the main reasons for the liking of unsweetened yogurt included “natural sourness”, while those for dislike were an “excessive sour taste”, “syneresis” and “dry aftertaste”. Similarly, the reasons for dislike of UMF18 yogurt were revealed by the comments such as “weird honey flavor”, “watery on top”, “minty off-flavour” and “yellowish tinge”, whereas those in regards to AMF15 yogurt were the “bitterness” and “astringent tastes”. The “weird honey flavor” described by some participants could be on account of manuka honey signature compounds such as methylglyoxal [16], methyl syringate and its novel glycoside, leptosin [44].

A high level of visible syneresis in the unsweetened control samples resulted in a significantly (*p* < 0.05) lower rating on the textural parameters of smoothness (4.37 ± 1.56) and mouthfeel (4.07 ± 1.63). All the sweetened samples had relatively high smoothness ratings (5 or above), but only the IS yogurts had mouthfeel acceptability ratings above 5 (5.10 ± 1.28). MB was accorded the highest hedonic rating among honey-sweetened yogurts for overall acceptability (5.05 ± 1.24), which could be due to the different flavour profiles of the honeys. In addition, the sensorial differences between the control and the yogurts sweetened with potentially prebiotic honey can be attributed to different concentrations of organic acids produced during fermentation and subsequent storage [3]. This concurs with the results of organic acid quantification (Figure 2), which showed that the levels of both lactic and acetic acids were higher in the sweetened honey samples than the control.

The addition of IS in yogurt improved participants’ acceptance for sweetness, sourness, and overall acceptance. All the manuka honey-sweetened yogurts were rated marginally lower than that with IS (5.32 ± 1.06). This outcome was similar to that found by Popa and Ustunol [43], in which the sensory characteristics of three varieties of honey-sweetened (7% *w*/*v*) yogurt had a lower overall acceptance than the sucrose-sweetened yogurt. Lower ratings were hypothesised possibly due to the yellow/amber colour of the honey masking that of typical strawberry yogurt and also due to the reduced viscosity of the honey-sweetened yogurts. Hansson et al. [45] postulated that the addition of invert syrup, which is generated through the hydrolysis of sucrose into equimolar amounts of glucose and fructose, results in more structured water molecules, thus decreasing the free water content (compared with sucrose at the same concentration). This phenomenon also reduced visual syneresis, a major factor for the dislike of yogurts, and was thus corroborated with higher overall acceptance of the IS sweetened yogurts.

### 3.4. Physicochemical Properties

#### 3.4.1. pH

The pH values significantly (*p* < 0.05) decreased during the storage period for all the yogurt types from day 7 onwards (Table 2). This can be ascribed to post-acidification, which is due to the continued production of organic acids in yogurts during the product shelf life by the starter culture bacteria [27]. Previous studies have also reported a similar decrease in the pH of yogurts during the extended cold storage [46]. A marginal (*p* > 0.05) increase in the pH observed for all the three honey-sweetened yogurts on day 21 can be explained by the production of biogenic amines and such metabolites. This phenomenon is initiated when the sugar substrates are consumed and the microbes have mostly protein sources available [31]. Nonetheless, the pH readings of all the yogurts in this study during the entire three weeks storage ranged from 4.0 to 4.5, which is considered to be acceptable from a product quality perspective.

#### 3.4.2. Colour

Colour of yogurt is a vital quality characteristic which influences the consumer liking in sensory evaluations. The addition of sweeteners to yogurts tended to increase the lightness values denoted by L*, more prominently (*p* < 0.05) in the case of IS (Table 2). A mild red hue was also observed in all the sweetened yogurt samples as emphasised by the low positive a* readings. Furthermore, positive b* values up to day 7 confirmed the yellow tinge of the honey yogurts, which was consistent with the visual sensory appearance. The honey-sweetened samples indeed recorded higher b* values than the unsweetened control, while the IS yogurt had the lowest b* readings. The observations in the L*, a* and b* readings with manuka honey addition in the present study are comparable to those reported by Mercan and Akın [2] for the yogurts sweetened with pine honey. However, no distinguishable pattern was perceived in the colour coordinates that will enable firm conclusions about the changes in the yogurt constituents during the three-week storage period.

#### 3.4.3. Apparent Viscosity

The unsweetened control samples exhibited extensive syneresis and behaved similarly to stirred yogurt, while all other samples were proper set-type yogurts. Due to the syneresis, the control samples showed low apparent viscosity (Table 2). The results concur with an increase in the apparent viscosity of set-type yogurts reported by Mercan and Akın [2] with the increasing levels of pine honey (up to 7%).

As can be seen in Table 2, the addition of IS to yogurts resulted in significantly (*p* < 0.05) higher apparent viscosity than manuka honey samples at the same concentrations (5% *w*/*v*). This can be explained by a stronger water-binding capacity of the monosaccharides (fructose and glucose) than the disaccharides such as sucrose [47]. Sucrose in invert syrup is hydrolysed to fructose and glucose, but honey additionally contains oligosaccharides, which will thus have a comparatively lower water-binding ability. This may have been one of the contributing factors in the high overall consumer acceptability of IS yogurts (Table 1).

The apparent viscosity of yogurt samples significantly (*p* < 0.05) decreased with the storage period, with the only exception being AMF15 yogurt, which also had the lowest value among all the sweetened yogurts (Table 2). In the case of AMF15 manuka honey yogurt, the viscosity was almost constant up to day 7, decreased on day 14, and increased towards the end of the storage period. Moreover, the net reduction in the viscosity of AMF15 yogurt (−285 cP) was lower than all the other sweetened yogurt samples, which was in the range of −1023.4 to −1293 cP (Table 2). A decrease in apparent viscosity of synbiotic yogurts supplemented with resistant starch during cold storage was reported by Donkor et al. [3], and the phenomenon was significantly affected by both the type of probiotic bacteria and the prebiotics under consideration. A steady decrease in apparent viscosity during storage was also observed by Aryana and McGrew [46] in probiotic *Lactobacillus casei* yogurts with differing chain length oligofructose and inulin. The researchers attributed this to the activity of bacterial enzymes on the casein micelle structure.

#### 3.4.4. Texture Analysis

The firmness of the yogurt samples containing sweeteners did not differ considerably (*p* > 0.05) from each other and had higher values than the unsweetened control sample throughout the observed storage period (Table 2). This was consistent with the hypothesis of Oliveira et al. [47] on the firmness being positively correlated to the total solids and protein contents. Towards the end of the refrigerated storage, most samples had higher firmness than the initial values (except UMF18). This increase in firmness was more prominent (*p* < 0.05) in the AMF15 and the unsweetened control samples. The phenomenon was reported in other studies, wherein the gel firmness increased with storage time [47,48,49]. Oliveira et al. [47] attributed this to gel structure reinforcement at low temperatures, which may be induced by the post-acidification process. As also hypothesised by Kailasapathy [50], post-acidification during the storage leads to casein rearrangement, which results in a more compacted and continuous structure.

The consistency values of all sweetened yogurts were considerably (*p* < 0.05) higher than the unsweetened sample for the entire storage days, except on day 21 (Table 2). A comparable effect observed in yogurts supplemented with inulin was ascribed to the structural network formed by the prebiotic and protein aggregates in yogurt gels [28,51]. In the present study the consistency values, similar to the observation for firmness, also increased up to day 14 and dropped slightly towards the end of the shelf life (except in the AMF15 and the unsweetened yogurts).

Cohesiveness values were not noticeably (*p* > 0.05) different among the sweetened samples throughout the storage days and were lower than the unsweetened control sample. It is interesting to note that the unsweetened samples had a high degree of visual syneresis, which correlates to high cohesiveness readings in yogurts [52]. The cohesiveness reduced during the storage period for all the yogurt samples (except IS and UMF18), and this decrease was apparent but not statistically significant (*p* > 0.05) for the unsweetened sample.

#### 3.4.5. Water Holding Capacity (WHC)

Yogurts were sufficiently (>90 ℃) heated during manufacture to induce the denaturation of whey proteins (heat-labile), so that they precipitate and coagulate along with casein micelles (heat-stable) on acidification [53,54]. This step is essential to ensure the yogurt gel stability, and thus, no major syneresis was observed while the sweetened yogurts were refrigerated in the containers. The protein gel network in our study was partially disrupted only when the yogurts were scooped out to serve the sensory participants.

WHC is an indicator of the ability of the protein matrix to hold free water [55] and is defined as the resistance to whey separation under applied force at high-speed centrifugation [53]. No statistical difference (*p* > 0.05) in the WHC values was observed among the sweetened yogurt types when tested at weekly intervals (Table 2). The sweetened yogurt samples recorded comparatively high WHC readings ranging from 65% to 72%, which slightly decreased (*p* > 0.05) during the storage period. This suggests the initial stability of the coagulated protein matrix, which partially degraded over the course of the storage [55]. The unsweetened control had a lower WHC owing to a weak yogurt gel structure; however, the values somewhat increased during the cold storage. Nevertheless, due to the aforementioned extensive syneresis in the control sample, the implications of adding sweeteners to probiotic yogurt on its physicochemical properties are not fully conclusive in this study.

Sert et al. [30] reported higher WHC values for sunflower honey yogurts than the present study, which increased with the concentration of honey (2–6% *w*/*v*). The results are not entirely comparable since, in addition to the different experimental conditions, no probiotic was incorporated in the yogurts in their research. Interestingly, an opposite trend during cold storage was observed in their study, with the WHC decreasing for the unsweetened control and increasing for the honey-sweetened yogurts. WHC can be affected by the prebiotic ingredient, and lower values were reported in synbiotic yogurts with *β*-glucan [56] and inulin [51].

## 4. Conclusions

Compared with the other varieties of manuka honey, AMF15 was more effective in maintaining the survival of *Lactobacillus reuteri* DPC16 in yogurt. However, the probiotic growth and survivability were highest with invert syrup (*p* < 0.05), which also had higher consumer acceptability in sensory trials. IS also had the highest apparent viscosity, which correlates with the overall sensory acceptance rating. Hence, IS can yield a commercially viable sweetened probiotic yogurt, but the combination cannot be termed as synbiotic since it contains only the simple sugars. Selection of a potentially synbiotic combination will entail a prebiotic component, such as the oligosaccharides present in honey (approximately 10%). The oligosaccharide constituents of the manuka honey will be further characterised in our subsequent research. Clinical studies on the in vivo physiological benefits for the host are also mandated to confirm a prebiotic effect.

The overall acceptance rating for all the sweetened yogurts was also positive with scores above 4.5 on a 7-point hedonic scale, which was significantly (*p* < 0.05) higher than the unsweetened control. Organic acids metabolite profile of honey yogurts, owing to a higher production of lactic and propionic acids, was more desirable than that with invert syrup or no added sweetener. The addition of honey can thus enhance both the functional health value and consumer acceptance of yogurts, which will be crucial since probiotic viability until the end of shelf life is a major concern in many of the commercially available yogurts. The probiotic counts in AMF15 manuka honey yogurt (7 log CFU/mL) after three weeks refrigerated storage were above the recommended dosage levels and significantly (*p* < 0.05) higher than the other honey yogurts (Manuka Blend and UMF18). AMF15 manuka honey yogurt can thus be further developed as an effective functional food by improving the physicochemical characteristics such as apparent viscosity. Further sensorial research can also include correlating the consumer acceptance studies and descriptive analysis to ascertain the drivers of liking for manuka honey yogurt.

## Figures and Tables

**Table 1:** Consumer panellist ratings (mean ± standard deviation) for the yogurts on a 7-point scale.

	Unsweetened Yogurt	Invert Syrup	Manuka Blend	Manuka UMF^TM^ 18^+^	Manuka AMF^TM^ 15^+^
Colour	5.04 ± 1.25 ^a^	5.30 ± 1.11 ^a^	5.24 ± 1.07 ^a^	5.11 ± 1.22 ^a^	5.30 ± 1.17 ^a^
Appearance	4.58 ± 1.41 ^a^	5.17 ± 1.19 ^b^	4.96 ± 1.02 ^ab^	5.09 ± 1.21 ^b^	5.10 ± 1.25 ^b^
Mouthfeel	4.07 ± 1.63 ^a^	5.10 ± 1.28 ^b^	4.60 ± 1.40 ^b^	4.87 ± 1.38 ^b^	4.92 ± 1.33 ^b^
Smoothness	4.37 ± 1.56 ^a^	5.36 ± 1.08 ^b^	5.01 ± 1.30 ^b^	5.37 ± 1.20 ^b^	5.27 ± 1.33 ^b^
Sweetness	3.51 ± 1.46 ^a^	4.82 ± 1.36 ^c^	4.80 ± 1.39 ^c^	4.49 ± 1.53 ^bc^	4.28 ± 1.45 ^b^
Sourness	3.73 ± 1.53 ^a^	4.95 ± 1.38 ^c^	4.86 ± 1.31 ^c^	4.44 ± 1.50 ^bc^	4.30 ± 1.45 ^b^
Overall Acceptance	4.34 ± 1.43 ^a^	5.32 ± 1.06 ^c^	5.05± 1.24 ^bc^	4.80 ± 1.44 ^bc^	4.65 ± 1.45 ^b^

Different superscripts in rows indicate a significant (*p* < 0.05) difference in sensory attributes for the yogurts.

**Table 2:** Physicochemical properties (mean ± standard deviation) of yogurts with three different honey and controls over the refrigerated storage period (21 days).

Yogurt	Storage (days)	pH	L*	a*	b*	Viscosity (cP)	Firmness (g)	Consistency (g.sec)	Cohesiveness (g)	WHC (%)
Unsweetened	1	4.42 ± 0.01 ^a,B^	88.5 ± 1.17 ^a,A^	−0.22 ± 004 ^a,A^	7.98 ± 0.45 ^c,A^	263.33 ± 45.020 ^a,A^	21.7 ± 7.0 ^a,AB^	370.7 ± 29.6 ^a,A^	0.72 ± 0.19 ^a,AB^	49.53 ± 11.32 ^a,A^
	7	4.11 ± 0.01 ^a,A^	89.8 ± 1.3 ^a,A^	−0.25 ± 0.33 ^a,A^	7.48 ± 0.37 ^b,A^	180.0 ± 17.890 ^a,B^	15.9 ± 2.5 ^a,A^	264.3 ± 116.2 ^a,A^	0.78 ± 0.070 ^a,B^	55.23 ± 5.89 ^a,A^
	14	4.12 ± 0.03 ^b,A^	95.6 ± 0.45 ^c,B^	5.00 ± 0.19 ^a,B^	−2.045 ± 0.39 ^b,B^	146.67 ± 31.410 ^a,B^	23.0 ± 5.3 ^a,AB^	419.0 ± 107.8 ^a,AB^	0.54 ± 0.060 ^b,AB^	57.02 ± 7.46 ^a,A^
	21	4.09 ± 0.02 ^b,A^	−	−	−	−	32.7 ± 4.5 ^a,B^	643.3 ± 144.1 ^a,B^	0.46 ± 0.060 ^a,A^	64.5 ± 5.19 ^a,B^
Invert Syrup	1	4.44 ± 0.01 ^b,B^	94.3 ± 0.77 ^c,A^	0.77 ± 0.02 ^b,A^	6.07 ± 0.06 ^a,A^	3805.0 ± 225.540 ^d,A^	37.3 ± 2.9 ^b,A^	761.3 ± 79.3 ^b,A^	0.50 ± 0.030 ^ab,A^	76.3 ± 5.25 ^b,A^
	7	4.14 ± 0 ^b,A^	93.2 ± 0.2 ^c,A^	0.78 ± 0.03 ^b,A^	6.67 ± 0.26 ^a,A^	3113.33 ± 146.65 ^d,B^	36.3 ± 2.5 ^b,A^	758.7 ± 42.4 ^b,A^	0.50 ± 0.020 ^b,A^	70.1 ± 8.0 ^b,A^
	14	4.13 ± 0.01 ^b,A^	96.03 ± 0.39 ^c,B^	5.11 ± 0.17 ^ab,B^	−3.46 ± 0.18 ^a,B^	2786.67 ± 142.36 ^d,C^	41.3 ± 0.6 ^c,A^	886.3 ± 69.2 ^c,A^	0.48 ± 0.010 ^ab,A^	71.9 ± 5.7 ^b,A^
	21	4.11 ± 0.03 ^b,A^	96.25 ± 0.14 ^c,B^	5.17 ± 0.11 ^a,B^	−3.19 ± 0.07 ^a,B^	2511.67 ± 187.87 ^b,D^	38.0 ± 3.6 ^a,A^	756.0 ± 138.7 ^a,A^	0.52 ± 0.060 ^a,A^	72 ± 4.4 ^b,A^
Manuka Blend	1	4.48 ± 0.01 ^c,C^	90.53 ± 0.5 ^ab,A^	1.53 ± 0.15 ^c,A^	7.2 ± 0.44 ^b,A^	3355.0 ± 95.650 ^c,A^	34.3 ± 8.1 ^b,A^	703.7 ± 217.2 ^b,A^	0.55 ± 0.120 ^ab,A^	72.44 ± 5.49 ^b,A^
	7	4.18 ± 0.02 ^c,B^	91.60 ± 0.2 ^b,A^	1.31 ± 0.02 ^c,A^	7.52 ± 0.24 ^b,A^	2723.33 ± 99.53 ^c,B^	38.0 ± 1.7 ^b,A^	795.3 ± 75.1 ^b,A^	0.48 ± 0.010 ^b,A^	71.2 ± 1.5 ^b,A^
	14	4.06 ± 0.01 ^a,A^	94.71 ± 0.36 ^b,B^	5.65 ± 0.13 ^c,B^	−2.32 ± 0.27 ^b,B^	2500.0 ± 116.62 ^c,C^	44.0 ± 1.7 ^c,A^	947.7 ± 92.7 ^c,A^	0.48 ± 0.010 ^ab,A^	71.7 ± 4.7 ^b,A^
	21	4.1 ± 0.06 ^a,A^	94.59 ± 0.21 ^b,B^	5.85 ± 0.10 ^c,B^	−1.97 ± 0.95 ^b,B^	2096.67 ± 52.79 ^a,D^	39.7 ± 2.1 ^a,A^	813.0 ± 51.0 ^a,A^	0.49 ± 0.030 ^a,A^	70.9± 2.7 ^b,A^
Manuka UMF^TM^ 18^+^	1	4.49 ± 0.01 ^c,C^	90.53 ± 0.5 ^ab,A^	2.09 ± 0.1 ^d,A^	9.55 ± 0.32 ^e,A^	3101.67 ± 264.530 ^c,A^	43.3 ± 2.1 ^b,A^	922.7 ± 87.2 ^b,A^	0.43 ± 0.030 ^b,A^	72.16 ± 4.31 ^b,A^
	7	4.19 ± 0.01 ^c,B^	90.50 ± 0.3 ^ab,A^	2.00 ± 0.12 ^d,A^	9.24 ± 0.26 ^d,A^	2750.0 ± 104.88 ^c,B^	37.0 ± 1.0 ^b,A^	810.0 ± 60.7 ^b,A^	0.47 ± 0.020 ^b,A^	70.4 ± 3.7 ^b,A^
	14	4.06 ± 0.02 ^a,A^	93.4 ± 0.7 ^a,B^	6.04 ± 0.12 ^d,B^	−0.69 ± 0.08 ^c,B^	2482.17 ± 114.23 ^c,C^	45.3 ± 1.5 ^c,A^	997.0 ± 46.0 ^c,A^	0.47 ± 0.010 ^a,A^	72.1 ± 2.9 ^b,A^
	21	4.08 ± 0.02 ^a,A^	93.66 ± 0.19 ^a,B^	6.28 ± 0.12 ^d,B^	−0.42 ± 0.09 ^d,B^	2078.33 ± 122.21 ^a,D^	37.0 ± 6.9 ^a,A^	803.3 ± 231.9 ^a,A^	0.52 ± 0.110 ^a,A^	68.8 ± 3.1 ^b,A^
Manuka AMF^TM^ 15^+^	1	4.44 ± 0.01 ^ab,C^	90.67 ± 0.3 ^ab,A^	1.29 ± 0.75 ^c,A^	8.77 ± 0.17 ^d,A^	2433.33 ± 149.480 ^b,A^	35.4 ± 2.2 ^b,AB^	682.7 ± 66.0 ^b,A^	0.55 ± 0.010 ^ab,A^	67.41 ± 3.66 ^b,A^
	7	4.2 ± 0.02 ^c,B^	91.0 ± 0.4 ^ab,A^	1.22 ± 0.64 ^c,A^	8.37 ± 0.17 ^c,A^	2426.67 ± 159.83 ^b,A^	33.3 ± 0.6 ^b,AB^	685.3 ± 39.3 ^b,A^	0.52 ± 0.010 ^b,A^	70.9 ± 1.5 ^b,A^
	14	4.09 ± 0.03 ^ab,A^	94.25 ± 0.15 ^b,B^	5.43 ± 0.13 ^bc,B^	−1.25 ± 0.24 ^c,B^	1628.33 ± 253.17 ^b,C^	32.3 ± 0.6 ^b,A^	619.3 ± 33.0 ^b,A^	0.48 ± 0.000 ^ab,A^	65.8 ± 4.4 ^b,A^
	21	4.17 ± 0.01 ^ab,B^	94.0 ± 0.22 ^a,B^	5.42 ± 0.09 ^b,B^	−1.03 ± 0.07 ^c,B^	2148.33 ± 146.89 ^a,B^	38.0 ± 3.6 ^a,B^	752.0 ± 105.2 ^a,A^	0.51 ± 0.060 ^a,A^	69.4 ± 6.4 ^b,A^

Different lowercase superscripts in columns indicate significant (*p* < 0.05) differences among the values for different yogurts (on that particular day). Different uppercase superscripts in columns indicate significant (*p* < 0.05) differences among the values during the storage period (for that particular yogurt). L*, a* and b* are the colour coordinates; WHC denotes the water holding capacity.

**Figure A1:**
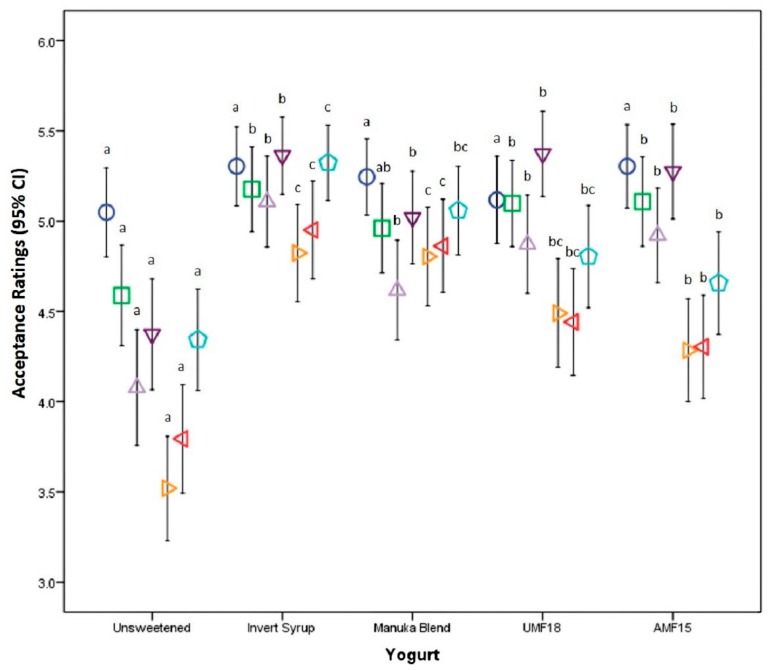
Error Bars of sensory acceptance ratings in 95% Confidence Intervals (CI) for yogurts with three different manuka honey and controls. Attributes: Colour (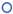), Appearance (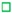), Mouthfeel (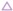), Smoothness (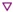), Sweetness (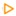), Sourness (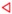); Overall acceptability (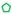). Different lowercase letters indicate significant differences (*p* < 0.05) in the sensory rating (across the different yogurt samples).
